# Secondary Buruli Ulcer Skin Lesions Emerging Several Months after Completion of Chemotherapy: Paradoxical Reaction or Evidence for Immune Protection?

**DOI:** 10.1371/journal.pntd.0001252

**Published:** 2011-08-02

**Authors:** Marie-Thérèse Ruf, Annick Chauty, Ambroise Adeye, Marie-Françoise Ardant, Hugues Koussemou, Roch Christian Johnson, Gerd Pluschke

**Affiliations:** 1 Swiss Tropical and Public Health Institute, Basel, Switzerland; 2 University of Basel, Basel, Switzerland; 3 Centre de Diagnostic et de Traitement de l'Ulcère de Buruli, Pobè, Benin; 4 Fondation Raoul Follereau, Cotonou, Benin; University of Tennessee, United States of America

## Abstract

**Background:**

The neglected tropical disease Buruli ulcer (BU) caused by *Mycobacterium ulcerans* is an infection of the subcutaneous tissue leading to chronic ulcerative skin lesions. Histopathological features are progressive tissue necrosis, extracellular clusters of acid fast bacilli (AFB) and poor inflammatory responses at the site of infection. After the recommended eight weeks standard treatment with rifampicin and streptomycin, a reversal of the local immunosuppression caused by the macrolide toxin mycolactone of *M. ulcerans* is observed.

**Methodology/Principal Findings:**

We have conducted a detailed histopathological and immunohistochemical analysis of tissue specimens from two patients developing multiple new skin lesions 12 to 409 days after completion of antibiotic treatment. Lesions exhibited characteristic histopathological hallmarks of Buruli ulcer and AFB with degenerated appearance were found in several of them. However, other than in active disease, lesions contained massive leukocyte infiltrates including large B-cell clusters, as typically found in cured lesions.

**Conclusion/Significance:**

Our histopathological findings demonstrate that the skin lesions emerging several months after completion of antibiotic treatment were associated with *M. ulcerans* infection. During antibiotic therapy of Buruli ulcer development of new skin lesions may be caused by immune response-mediated paradoxical reactions. These seem to be triggered by mycobacterial antigens and immunostimulators released from clinically unrecognized bacterial foci. However, in particular the lesions that appeared more than one year after completion of antibiotic treatment may have been associated with new infection foci resolved by immune responses primed by the successful treatment of the initial lesion.

## Author Summary

Buruli ulcer (BU) is a chronic necrotizing skin disease presenting with extensive tissue destruction and local immunosuppression. Standard treatment recommended by the WHO includes 8 weeks of rifampicin/streptomycin and, if necessary, wound debridement and skin grafting. In some patients satellite lesions develop close to the primary lesion or occasionally also at distant sites during effective antibiotic treatment of the primary lesion. We performed a detailed analysis of tissue specimens from lesions that emerged in two BU patients from Benin 12 to 409 days after completion of chemotherapy. Histopathology revealed features of tissue destruction typically seen in BU and degenerated acid-fast bacilli. In addition, lesions contained organized immune infiltrates typically found in successfully treated BU lesions. Secondary lesions emerging many months after completion of chemotherapy may have been caused by immune response-mediated paradoxical reactions. However, the late onset may also indicate that they were associated with new infection foci spontaneously resolved by adaptive immune responses primed by antibiotic treatment of the primary lesions.

## Introduction

Buruli ulcer (BU) is a chronic necrotizing infection of subcutaneous tissue caused by *Mycobacterium ulcerans* [1]-[4]. BU seems to start usually as a movable subcutaneous nodule or papule and may later progress to a plaque or edema. After destruction of subcutaneous tissue, the skin may break down centrally leading to the development of largely painless necrotic skin ulcers with characteristic undermined edges. These may progress to large necrotic lesions. *M. ulcerans* is unique among mycobacterial pathogens in that it resides in advanced lesions mainly extracellularly. A histopathological hallmark of progressing BU is a poor local inflammatory response in the presence of clusters of extracellular acid-fast bacilli surrounded by areas of necrosis [5]–[7]. *M. ulcerans* produces a toxin with a polyketide-derived macrolide structure, named mycolactone, which plays a central role in tissue destruction and local immunosuppression. Observations both in cell culture and infection models indicate that cells infiltrating BU lesions are killed due to the cytotoxic and apoptosis inducing activity of mycolactone [7]–[10]. While *M. ulcerans* may be captured by phagocytes during initial stages of infection, it appears to persist only transiently inside these host cells [11], [12]. After killing of the phagocytes, extracellular growth leads to the development of extracellular mycolactone-producing bacterial foci in areas of coagulating necrosis. Thermosensitivity of *M. ulcerans* seems to favor development of skin lesions of the limbs [13]–[15].

Clinical diagnosis of BU can be confirmed by insertion sequence 2404 (*IS2404*) PCR [16]–[18], microscopic detection of acid-fast bacilli (AFB), culture of *M. ulcerans* [19] and histopathological examination of lesions [6], [20]–[22]. While surgery has traditionally been the only recommended treatment for BU [23], [24], WHO recommends currently as a first-line treatment a combination therapy with rifampicin and streptomycin (R/S) for eight weeks for all forms of the active disease [25], [26]. After a pilot study assessing treatment of BU with R/S [25], a case-series in Benin showed that of 224 patients 215 were successfully treated [27], with 47% of them receiving antibiotics only. More recently, studies by Nienhuis et al., Kibadi et al. and Sarfo et al. [28]–[30] reconfirmed efficacy of R/S treatment. However, débridement, surgery and skin grafting may be used as an adjunct to the antimicrobial therapy, mainly to remove necrotic tissue, cover skin defects and correct deformities.

Reported rates of recurrence after surgical treatment alone range between 6% and 47% because even wide surgical excision of lesions may not remove all bacilli [31]–[34]. Recurrences may be caused by small numbers of *M. ulcerans* that have spread to healthy tissue surrounding the primary lesion [5]. Also lymphohematogenous spread of the mycobacteria may occur, since subsets of BU patients develop multiple skin lesions or metastatic osteomyelitis [35]–[39]. Although clinical trials indicate that some bacilli may survive the recommended eight week course of antibiotic treatment [28], [30], recurrence rates after R/S treatment are as low as 1–2% [27], [29].

In active BU disease, a protective cloud of mycolactone around the mycobacterial clusters is thought to both destroy infiltrating leukocytes and hinder them from passing pro-inflammatory signals to other cells. It is most likely, but still remains to be formally proven, that mycolactone production is reduced or abolished early after the onset of R/S chemotherapy due to impairment of mycolactone synthesis, bacterial growth arrest and/or bacterial cell death, reflected by ‘beaded’ appearance of AFBs (MT Ruf; unpublished results). Declining toxin levels allow leukocytes to reach the extracellular mycobacteria, leading to their phagocytosis and destruction [40]. Chronic leukocyte infiltration cumulates in the development of ectopic lymphoid structures [20]. After eight weeks of R/S chemotherapy, antigen presenting cells as well as B and T lymphocyte foci are found in large numbers inside the BU lesions [20] indicating that antigen recognition and processing is leading to active *M. ulcerans* specific immune responses. Vigorous local immune responses during R/S treatment may lead in some of the patients to the development of clinical deteriorations, ‘paradoxical reactions’ [41]. For this study we conducted detailed immunohistochemical analyses of secondary lesions which had occurred at extended periods of time after effective R/S treatment at different body sites.

## Materials and Methods

### Ethics statement

Ethical approval for analyzing patient specimens was obtained from the ethical review board of the Ministry of Health of Benin. Written informed consent from the guardians of the patients was obtained before surgical specimens were used for reconfirmation of BU as well as a detailed immunohistological analysis.

### Study participants

Both patients, two six year old boys, included in this study were laboratory-confirmed BU cases with one primary lesion. Both received a combination of rifampicin (10 mg/kg body weight) and streptomycin (15 mg/kg body weight) administered daily over 8 weeks at the Centre de Diagnostic et de Traitement de l'Ulcère de Buruli (CDTUB) in Pobè, Benin according to the WHO recommendations. Both patients developed several new lesions at different parts of the body, 12 – 409 days after completion of antibiotic treatment. These lesions were removed by limited excision and no additional antibiotic treatment was administered. Excised tissue from a number of these new lesions became available for histopathological analysis (Table 1). Both patients were tested negative for HIV, shistosomiasis, hepatitis B and syphilis. Blood values tested and the nutritional status was within the limits typically found in children in rural Africa. Only patient 2 presented with a BCG scar.

**Table 1:** Features of skin lesions that emerged after completion of antibiotic treatment.

Patient	Primary lesion	Time span (days) between completion of antibiotic treatment and occurrence of secondary lesion	Time span (days) between occurrence of secondary lesion and surgical excision	Nature of satellite lesion	Location of satellite lesion	Distance of satellite from primary lesion
1	ulcer at the right upper arm/elbow	75	4	ulcer 1	right axilla	10 cm
		275	1	**nodule 1**	back	25 cm
		409	2	**ulcer 2**	right shoulder	20 cm
		409	2	**nodule 2**	thorax	30 cm
2	ulcer at the right upper leg/knee	12	6	nodule 1	right upper leg	5 cm
		46	33	**nodule 2**	right lower leg	15 cm
		54	25	**nodule 3**	right upper leg	5 cm
		176	1	nodule 4	right foot	30 cm

Lesions from which tissue samples became available for histopathological analysis are written in bold letters.

### Histopathological analysis

Tissue specimens analyzed are listed in Table 1. Samples were fixed in 4% neutral-buffered paraformaldehyde for 24 h and subsequently transferred to 70% ethanol for storage and transport. Afterwards biopsies were dehydrated, embedded in paraffin, and cut into 5 µm thin sections. After deparaffinization and rehydration sections were either directly stained with haematoxylin/eosin (HE) or Ziehl-Neelsen/methylenblue (ZN) according to WHO standard protocols [42] or further processed for immunohistochemistry (IHC). For IHC antigen retrieval was performed according to standard protocols either with citrate buffer, EDTA buffer or by enzymatic trypsin digestion (Dako® Education guide: Immunohistochemical Staining methods). Afterwards endogenous peroxidase was inactivated with 0.3% H_2_0_2_ for 20 min and prevention of unspecific binding was achieved by incubation with blocking serum matching the secondary antibody host. Primary antibodies specific for N-Elastase (polymorphonuclear neutrophils [PMNs]; Dako clone NP57), CD3 (T-lymphocytes; Dako clone F7.2.38), CD8 (CD8^+^ T-lymphocytes; Serotec clone 4B11), CD14 (Monocytes/macrophages; Novocastra clone 7) and CD20 (B-lymphocytes; Novocastra clone7D1) were appropriately diluted in phosphate buffered saline (PBS) containing 0.1% Tween-20 and added to the slides for 1 h at room temperature or over night at 4°C. After incubation with a matching biotin-conjugated secondary antibody staining was performed using the Vector NovaRED system. Haematoxylin was used as a counter stain.

## Results

### Clinical presentation of BU patients developing secondary skin lesions after completion of antibiotic treatment

In the present report we describe clinical and histopathological observations in two BU patients that have developed series of new skin lesions (Table 1) after effective anti-mycobacterial chemotherapy.

**Figure 1:**
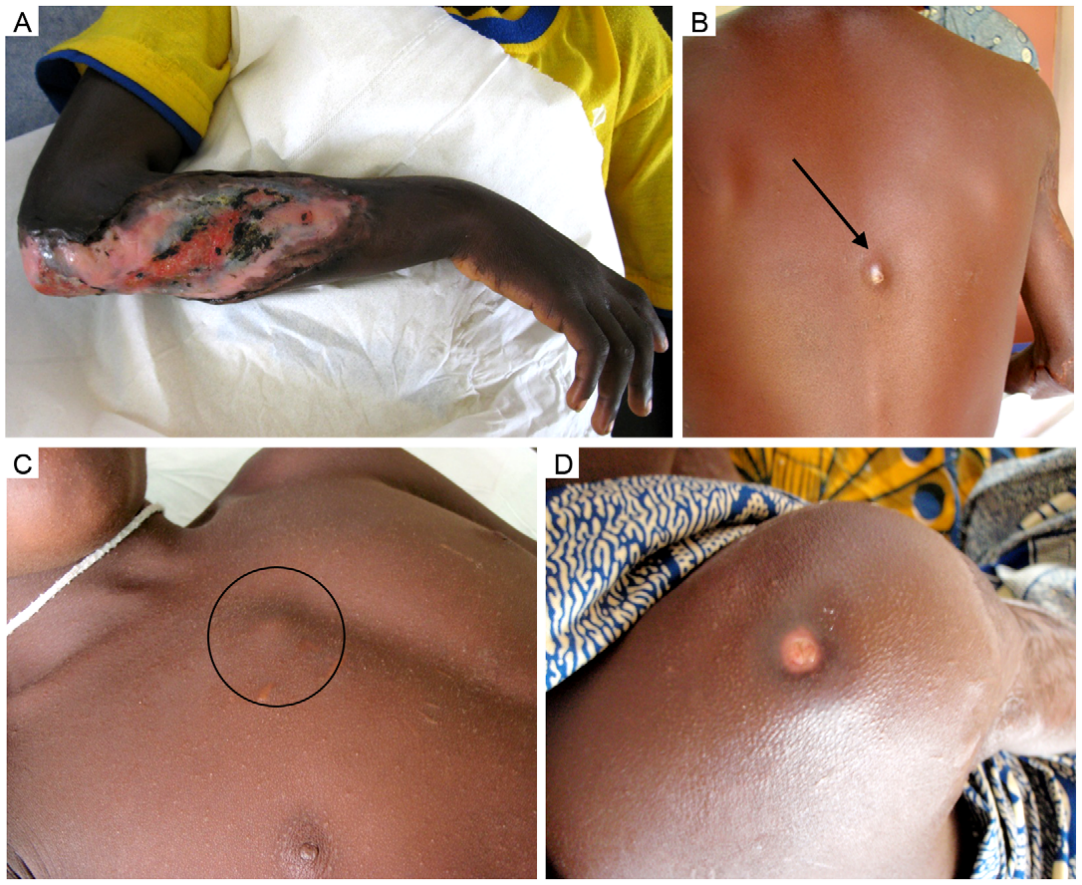
Clinical presentation of lesions (Patient 1). A: Initial ulcerated lesion at the right arm, reaching from the elbow to the forearm. B: Nodule1 appearing on the back, 275 days after end of antibiotic treatment. Both, nodule 2 on the thorax (C) and an ulcerated plaque on the right shoulder (D) had appeared 409 days after completion of antibiotic treatment.

Patient 1, a six year old boy, presented at the Centre de Diagnostic et de Traitement de l'Ulcère de Buruli (CDTUB) in Pobè, Benin with a 15×15 cm ulcerated plaque lesion at the right forearm and elbow with undermined edges characteristic for BU (Figure 1A). First BU symptoms had been noticed eight month before and the lesion had been treated afterwards with traditional medication. After admission to the hospital clinical diagnosis was confirmed by a positive *IS2404* PCR result of a fine needle aspirate, whereas culture was negative. As recommended in the WHO guidance on the role of specific antibiotics in the management of BU [26] the patient received for 8 weeks daily oral rifampicin (10 mg/kg body weight) and intramuscular streptomycin (15 mg/kg body weight). 37 and 65 days after start of this standard R/S chemotherapy wound débridement was performed, 18 days after the last excision skin grafting was done and 83 days after grafting the primary lesion had healed.

75 days after completion of chemotherapy a first new ulceration 0,5×0,5 cm (ulcer 1) in the axilla of the right arm emerged. After performing some débridement, this lesion had healed 35 days later and the patient was discharged from hospital. 275 days after completion of chemotherapy the patient was readmitted with a non ulcerated fluctuant nodule 1,5×1,5 cm (nodule 1) on the back (Figure 1B), which was excised with primary skin closure one day later. 409 days after completion of chemotherapy two more lesions developed, a 1,5×1,5 cm nodule (nodule 2) on the thorax (Figure 1C) and an ulcerated plaque 3×3 cm on the right shoulder (ulcer 2) (Figure 1D). Both lesions were excised two days after admission. From both lesions specimen taken were *IS2404* PCR as well as AFB positive, whereas culture was negative. 28 days after the surgical intervention, the patient was discharged from hospital. No further relapses were observed after 10 months of follow-up (February 2011).

Patient 2, also a six year old boy, presented at the CDTUB with a 20×15 cm ulcerated lesion on the interior side of his right upper leg and knee. Undermined edges as well as ‘cotton wool’ appearance of necrotic tissue at the center of the lesion were characteristic for BU [42]. Clinical diagnosis was confirmed by positive *IS240*4 PCR results and microscopic detection of AFB in swab samples. Surgical débridement was performed 29 days after start of standard R/S chemotherapy followed 10 days later by skin grafting. Twelve days after completion of antibiotic treatment, a nodule (nodule 1) 2×2 cm; had emerged about 5 cm proximal of the border of the primary lesion at the upper right leg and was excised 7 days later. The initial lesion as well as the lesion at the excision site had healed 39 days after completion of the antibiotic treatment (i. e 57 days after skin grafting) and the patient was discharged from hospital.

One week after discharge (46 days after completion of antibiotic treatment) the patient was readmitted with a second nodule (1,5×1,5 cm) located at the lower right leg about 15 cm distal of the border of the primary lesion. Again eight days later (54 days after completion of antibiotic treatment) a third nodule (nodule 3) (3×2 cm) had emerged at the upper right leg located 5 cm proximal of the initial wound. These two nodules were excised 93 days after completion of the antibiotic therapy. While AFB staining, as well as *IS2404* PCR confirmed the presence of *M. ulcerans,* both nodules were culture negative.

After surgical excision and healing of the satellite lesions the patient was discharged, but re-admitted 176 days after completion of antibiotic treatment with a fourth nodule (nodule 4) 2×2 cm on the right foot. A minimal surgical intervention was performed and the patient was discharged 10 days later and no further relapses were observed after 10 months of follow-up (February 2011).

### Histopathological features of excised secondary lesions

Histopathological and immunohistochemical analyses were performed with nodule 1, nodule 2 and ulcer 2 from patient 1, and nodule 2 and 3 of patient 2 (Table 1). These lesions appeared 275 to 409 days and 46 to 54 days, respectively, after completion of chemotherapy. Analysis yielded comparable results for all specimens analyzed. Typical data are shown below.

**Figure 2:**
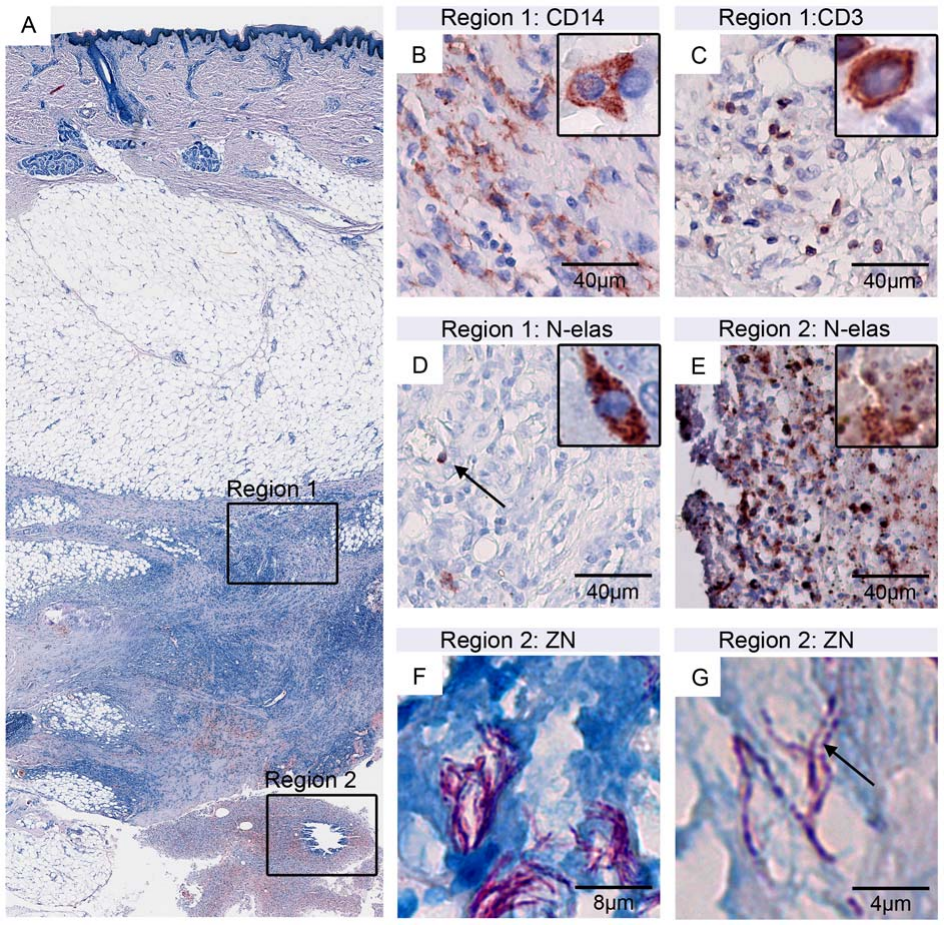
Histopathological presentation of secondary lesions. Histological sections (nodule 2 of patient 2) were stained either with Ziehl-Neelsen (counterstain methylenblue; A, F, G) or with antibodies against cell surface or cytoplasmic markers (counterstain haematoxylin; B–E). A: Overview over excised tissue specimen revealing typical BU pathology features like fat cell ghosts, necrosis, epidermal hyperplasia and AFB (region 2) as well as a strong mixed infiltration typically observed in successfully treated BU lesions (region 1). B: CD14 staining of macrophages/monocytes; C: CD3 staining of T-cells; D: Elastase staining of neutrophils. In the necrotic region 2 large numbers of elastase-positive neutrophilic debris (E) and small clumps of AFB (F) with a beaded appearance (G) were observed.

Features characteristic for BU pathology, such as fat cell ghosts, necrotic soft tissue, hemorrhages, and epidermal hyperplasia were present in all specimens analyzed. As shown in Figure 2A, necrotic areas were massively infiltrated with leucocytes, a feature, which is characteristic for successfully treated lesions [20]. Immunohistochemical analysis revealed mixed cellular infiltrations (Region 1) composed of large numbers of CD14 positive macrophages/monocytes (Figure 2B) and CD3 positive T-cells (Figure 2C). In contrast, intact N-elastase positive neutrophils were rare (Figure 2D). As described previously [20], some areas, such as the AFB containing region 2 in Figure 2A contained N-elastase positive debris (Figure 2E), which appears to represent remains of an early wave of neutrophilic infiltration. ZN staining revealed AFBs and globi like structures in the necrotic areas in the tissue from nodule 1 of patient 1 and in nodules 2 and 3 of patient 2 (Figure 2F, G). AFBs had a ‘beaded’ appearance (Figure 2G), which has been shown to be an indicator for loss of viability in the case of *M. leprae* [43].

**Figure 3:**
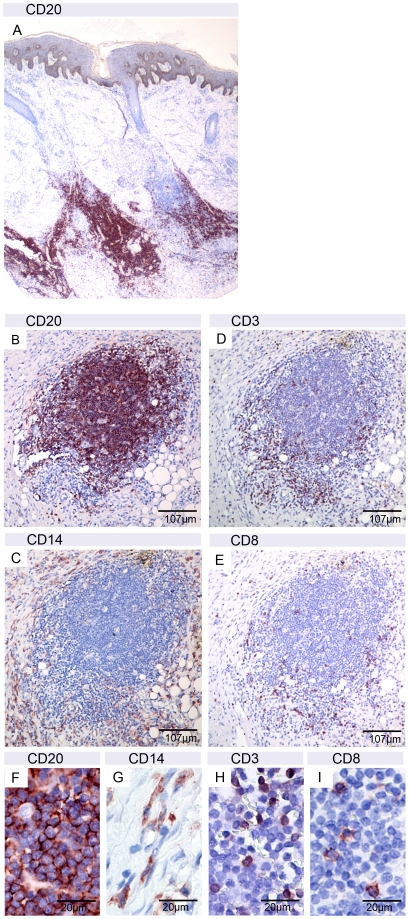
Presence of B-cell clusters in the secondary lesions. A: Band of CD20 positive B-cells in sections of ulcer 2 of patient 1. B–E: serial sections of nodule 3 of patient 2 with a small dense cluster of CD20 positive B-cells (B) surrounded by CD14 positive macrophages/monocytes (C) and few interspersed CD3 positive T-cells (D) from which the majority was CD8 negative (E). Higher magnification (F–I) revealed a very dense package of the B-cells.

Clusters of CD20 positive B-cells, another hallmark of ectopic lymphoid tissue developing in BU lesions after successful treatment [20], were also found in the tissue specimens analyzed. These clusters varied in size ranging from very large accumulations forming a band throughout the whole tissue (Figure 3A) to small dense B cell accumulations (Figure 3B) surrounded by CD14 positive macrophages/monocytes (Figure 3C) and few interspersed CD3 positive T-cells (Figure 3D), mostly CD8 negative (Figure 3E), mainly at the border of the dense B-cell cluster. Higher magnifications confirmed the dense packaging of B-cells (Figure 3F) and more dispersed distribution of other leucocytes (Figure 3G–H).

**Figure 4:**
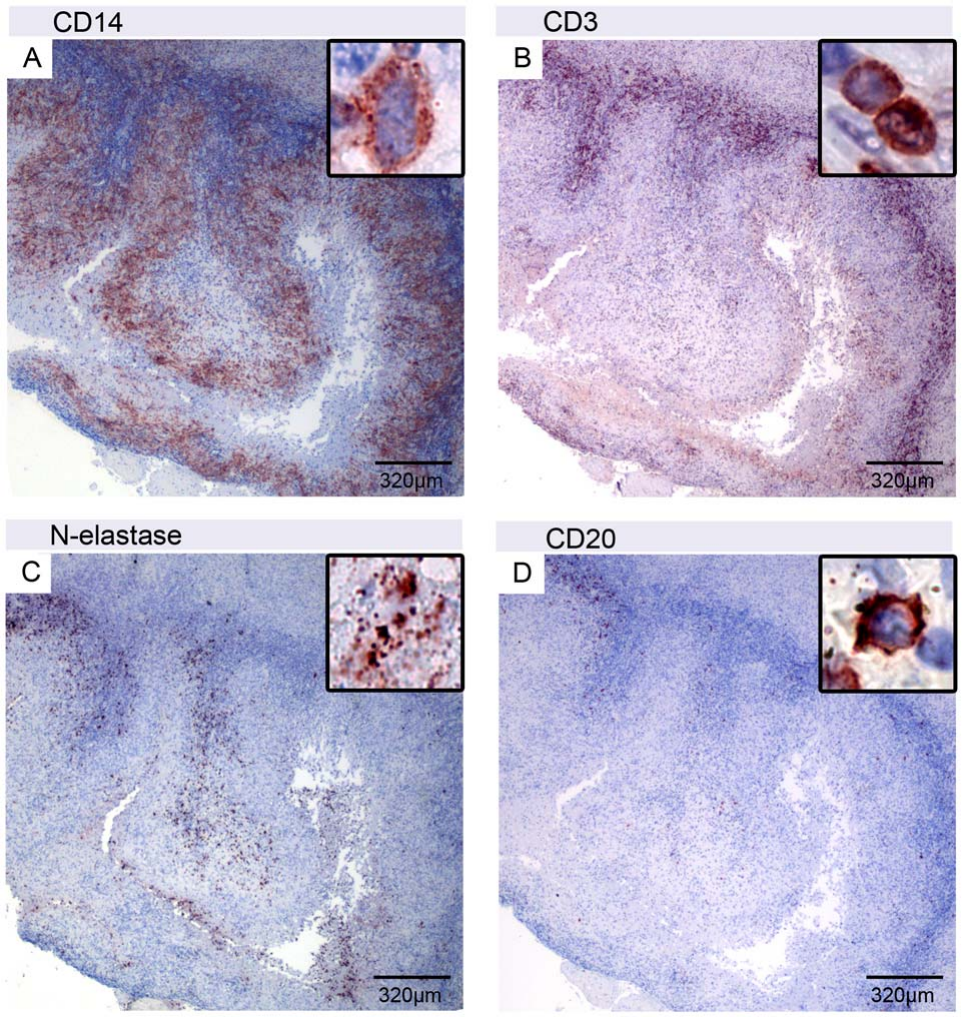
Bands of leucocytes surrounding an uninfiltrated necrotic area. Serial sections of nodule 2 of patient 1 with a necrotic area surrounded by a belt of CD14 positive monocytes/macrophages (A) and a more external second belt of CD3 positive T-cells (B). The necrotic core contained N-elastase positive neutrophilic debris (C), but no intact neutrophils (D insert). Clusters of CD20 positive B-cells were found away from the necrotic core (D).

In some parts of the lesions small uninfiltrated necrotic areas surrounded by belts of leucocytes still remained. Immunohistochemical analyses gave indications for sequential infiltration of these areas by different types of leucocytes (Figure 4). Necrotic areas were surrounded by an inner dense belt of CD14 positive macrophages/monocytes (Figure 4A), which thus seem to constitute the first line of infiltration after decline of cytotoxic mycolactone levels. While a belt containing large numbers of CD3 positive T-cells representing a second line of infiltration were found in direct neighborhood to the macrophages (Figure 4B), intact N-elastase positive neutrophils (Figure 4C) and CD20 positive B-cells (Figure 4D) were comparatively rare in these settings. However a strong staining of N-elastase positive neutrophilic debris was observed inside the necrotic areas (Figure 4C). Higher magnification revealed no intact cells in this location (Figure 4C insert).

## Discussion

In this report we describe the development of series of new skin lesions in two BU patients 12 – 409 days after completion of antibiotic treatment. The newly emerging nodules and ulcerations were located either at some distance from the initial lesion at the same extremity or at other body locations. Detection of *M. ulcerans* DNA by *IS2404* PCR, microscopic detection of AFBs and the presence of histopathological features characteristic for BU demonstrated that the new lesions were associated with *M. ulcerans* infection. Degenerated appearance of the AFBs and the presence of massive immune cell infiltrates in most parts of the lesions were on the other hand characteristic for treated BU lesions [20].

Detailed immunohistochemical analyses showed that residual necrotic areas were surrounded by an outer belt of T-lymphocytes and an inner belt of macrophages/monocytes with appendices reaching into the necrotic tissue. These belts of intact leucocytes seem to reflect ongoing efforts of the immune system to resolve the necrotic areas. In contrast, remains of neutrophils found inside the necrotic areas seem to be leftovers of early acute neutrophilic infiltration waves. These are also observed in early phases of *M. ulcerans* infection in experimentally infected mice (MT Ruf et al., unpublished results). Apart from these residual necrotic regions, the destroyed adipose and dermal connective tissue layers showed angiogenesis and contained abundant leukocyte infiltrates. It is thought that such chronic infiltrates can only develop once the concentration of the cytopathic *M. ulcerans* macrolide toxin mycolactone has diminished [20], [44]. Imbedded in the diffuse infiltrates, more structured leukocyte accumulations, such as B-cell clusters indicative for humoral immune responses [45]–[47] and first granulomas were found. In BU granulomas may function primarily as a place for antigen presentation and adaptive immune response, rather than for sequestration of the mycobacteria [20].

Recently O'Brien et al have described the occurrence of paradoxical reaction in two Australian BU patients during R/S treatment of BU [41]. After a first clinical improvement worsening of the clinical appearance occurred. For one patient incomplete excised wound margins showed paradoxical reaction whereas for the other patient a more distant secondary lesion opened, before end of treatment was reached. Worsening of lesions motivated a change in the treatment regimen and additional surgery. After detailed evaluation, data have been interpreted as immune-mediated reactions rather than treatment failures, as it has been shown that antibiotic therapy for *M. ulcerans* leads to a reversal of local immunosuppression [20], [41], [48]. The observed vigorous local immune responses are most likely caused by bacterial antigens and immunostimulators released from the killed mycobacteria. Similar paradoxical reactions have been well described for *M. tuberculosis*, *M. leprae* and in particular in immunocompromised HIV patients who commence HAART [49]–[52]. In tuberculosis an elevation of the TNF-α level, stimulated by lipoarabinomanan and other lipopolysaccharides present in the cell wall, has been postulated as an initial step in the development of paradoxical reaction [53], [54]. Limited surgical excision may help to resolve paradoxical reactions by reducing the burden of mycobacterial antigens and in some clinical settings corticosteroids have been used for down regulation of immune responses [55]–[58].

In the case of the two patients described here, new lesions developed at prolonged periods of time after completion of antibiotic treatment. These lesions may represent secondary *M. ulcerans* infection foci that were already present without clinical signs and symptoms during antibiotic treatment and development of new lesions may be the consequence of delayed paradoxical reactions. However, in particular the lesions that appeared more than one year after completion of antibiotic treatment may also have been associated with new infection foci caused by new *M. ulcerans* infections or by mycobacteria that had survived the eight week course of R/S treatment [28], [29]. These may have been resolved by immune responses primed by the successful treatment of the primary lesion. If this is the case, detailed analysis of immune responses in more patients developing such secondary lesions may provide important insights into immune protection against *M. ulcerans* and support vaccine design [59].
